# Serum anti-mullerian hormone levels and age among Samoan women

**DOI:** 10.1186/s12958-025-01379-y

**Published:** 2025-03-19

**Authors:** Grace O’Brien, Geralyn Lambert-Messerlian, Nicola L. Hawley, Ulai T. Fidow, Take Naseri, Muagututi‘a Sefuiva Reupena, Erin E. Kershaw, Marinelle B. Azar, Martha M. Pangburn, Stephen T. McGarvey

**Affiliations:** 1 Department of Epidemiology and International Health Institute, Brown University School of Public Health, Providence, Rhode Island, USA; 2 Departments of Pathology and Laboratory Medicine and Obstetrics and Gynecology, Women and Infants Hospital, The Alpert Medical School at Brown University, 70 Elm Street, 2nd floor, Providence, Rhode Island 02903 USA; 3 Department of Chronic Disease Epidemiology, Yale School of Public Health, New Haven, Connecticut, USA; 4 Department of Obstetrics and Gynecology, Tupua Tamasese Meaole Hospital, Ministry of Health, Government of Samoa, Apia, Samoa; 5 Ministry of Health, Government of Samoa, Apia, Samoa; 6 Lutia i Puava ae Mapu i Fagalele, Apia, Samoa; 7 Division of Endocrinology and Metabolism, Department of Medicine, University of Pittsburgh, Pittsburgh, PA USA; 8 Department of Anthropology, Brown University, Providence, Rhode Island USA

**Keywords:** AMH, Ovarian reserve, Body mass index, Polynesian, Obesity, Polycystic ovary

## Abstract

**Background:**

Serum AMH levels in adult women are part of the diagnostic criteria for polycystic ovary syndrome (PCOS), a condition with marked infertility and metabolic risks. Yet, little is known about AMH levels among women from ethnic minority populations, especially its associations with age and obesity. The objective is to describe the association of age and serum anti-mullerian hormone (AMH) among Samoan women, provide age specific AMH reference levels, and examine the associations of AMH with adiposity and reproductive factors.

**Methods:**

A cross-sectional, retrospective study of a representative community-based sample from Samoa was conducted. 670 women with no known reproductive disorders, reproductive surgeries, or hormonal contraceptive use, age 25–51 years, were included. Adiposity was assessed by body mass index (BMI) using Polynesian-specific criteria for obesity. Serum AMH was determined by enzyme-linked immunosorbent assay. Serum total testosterone and sex hormone binding globulin were measured, and the free androgen index was calculated. Hormonal contraceptive use, menstrual regularity, and tobacco use were assessed by questionnaire. PCOS prevalence was estimated using current guidelines.

**Results:**

Despite a high prevalence of obesity and overweight in Samoan women, serum AMH and its age related decline were similar to those reported in other populations. AMH was negatively associated with age. AMH decline with age in Samoan women is best described by a cubic model. AMH was not associated with BMI or insulin resistance. PCOS prevalence was estimated at 3.4–5.1%.

**Conclusion:**

This study was the first to construct an age specific AMH reference range for Samoan women. PCOS prevalence appears low, supporting other published studies that have demonstrated a complex relationship between adiposity and reproductive health in Samoan women.

**Clinical trial number:**

Not applicable.

**Supplementary Information:**

The online version contains supplementary material available at 10.1186/s12958-025-01379-y.

## Background

Polycystic ovary syndrome (PCOS) is a condition associated with infertility and multiple lifelong health comorbidities [1, 2]. Individuals clinically diagnosed with PCOS experience increased risk for insulin resistance, overt type 2 diabetes, dyslipidemia, hypertension, cardiovascular disease, as well as breast and endometrial cancers. Complications in pregnancy, such as pre-eclampsia, recurrent loss, fetal macrosomia, and perinatal mortality are also higher in women with PCOS than without. Mental health morbidity, such as anxiety and depression, is more frequent.

While PCOS has serious long term health consequences, diagnosis is challenging, especially across different ethnic groups [3, 4]. There are several clinically accepted definitions for the condition, including those promulgated by the NIH (1990), Rotterdam (2003) or AE-PCOS Society (2006). In 2023, an international evidence-based guideline was published to promote consistency in PCOS diagnosis [5]. The group consensus requires two of three features for diagnosis of PCOS: clinical or biochemical hyperandrogenism, ovulatory dysfunction (i.e., oligo- or amenorrhea), and either high antral follicle count on ovarian ultrasound or elevated serum anti-mullerian hormone (AMH). Measurement of serum AMH levels is newly supported as an acceptable surrogate for antral follicle count by ultrasound in adult women. This is particularly helpful in low resource settings where ultrasound is not accessible.

Despite the widespread interest and utility of AMH as a biomarker for ovarian reserve, its use in clinical practice remains limited. One critical reason is the lack of normative ranges across different populations. Most studies have been performed in higher income countries with participants of European ancestry or ethnicity, and there is still much unknown about individual and population variation in AMH. The rate of decline in AMH with age appears to vary by ancestry or ethnicity [6, 7]. For example, a cross-sectional study of American women demonstrated that Black, White, Chinese, and Latina women have different rates of AMH decline with age. Using adjusted multivariate models, Black women had lower levels of AMH at younger ages, and their age associated AMH decline was slower compared to White, Latina, and Chinese women. AMH levels are also influenced by reproductive, lifestyle, and biological characteristics [8–10]. For example, most studies report reduced AMH with cigarette smoking, oral contraceptive use, physical activity, and increased body mass index [11]. This knowledge gap is particularly large in high-risk populations that are historically underrepresented in science.

Contemporary Samoan populations have high levels of adiposity and cardiometabolic risk, and this risk is increasing over time, in part, due to changes in dietary and physical activity stemming from the nutrition transition [12, 13]. A 2010 national survey revealed that over 91% of Samoan women were overweight or obese based on Polynesian body composition references, making Samoa one of the nations with the greatest prevalence and severity of obesity [12]. Obesity and PCOS frequently co-occur leading one to hypothesize a high prevalence of PCOS in Samoa. Increased adiposity is associated with changes in sex steroid metabolism, androgen production, and SHBG levels, all of which impact the presentation of PCOS [14]. There is currently no AMH reference range for women of Samoan or other Polynesian ancestry to aid in PCOS diagnosis.

The aims of the present study are to describe AMH levels among Samoan women, provide age specific AMH reference levels, and examine the associations of AMH with adiposity and reproductive factors. These data could facilitate PCOS diagnosis in a community where routine ovarian ultrasound is lacking and/or clinical assessment is not commonly performed. This information is crucial for identifying women at an elevated risk of developing cardiovascular disease related to PCOS diagnosis. This report will also extend the limited knowledge on AMH levels from understudied and underserved ethnic populations.

## Methods

### Study population

These data are from a cross-sectional, population-based genome wide association study (GWAS) of adiposity and cardiometabolic risk conducted in Samoa in 2010. Participants aged 25 to 65 years who self-reported that all four grandparents were Samoan were recruited. Anthropometry, blood pressure, fasting serum, and reproductive, general health and cigarette and alcohol use questionnaire measures were conducted among > 3,000 adults from 33 villages [12].

### Study sample

Women 25–51 years of age were eligible if they had no history of reproductive surgical procedures or hormonal contraceptive use. In addition, participants were excluded if they had missing fasting serum glucose and insulin measures, reproductive biomarkers, or menstrual cycle regularity information. The Brown University Institutional Review Board and the Health Research Committee of the Samoan Ministry of Health approved the parent research protocol and informed consent process. The study, data collection protocols, and participant rights were explained verbally in Samoan by trained fieldworkers. All participants gave written consent in the Samoan language.

### AMH measurement

Serum AMH levels were measured by enzyme-linked immunosorbent assays (picoAMH or ultrasensitive AMH, Ansh Labs, Webster, TX). Inter- and intra-assay coefficients of variation were less than 15%. For women 20–39 years, we used the ultrasensitive AMH assay with a limit of detection (LoD) of 0.08 ng/mL. For older women, we used the picoAMH assay which is more sensitive with a LoD of 0.006 ng/mL. AMH values that were < LoD were imputed with 0.005 ng/mL. The results of the ultrasensitive and picoAMH assays are comparable [15].

### Androgen and cardiometabolic measures

Serum total testosterone and sex hormone binding globulin (SHBG) were measured by automated chemiluminescent immunoassays (Siemens, Los Angeles, CA) as previously described [13]. Total testosterone values that were below the limit of detection (LoD, 20 nmol/L), were imputed with 19 nmol/L. The free androgen index (FAI) was calculated as (total testosterone x 100)/SHBG and has been shown to be an indicator of androgen levels in the context of women’s reproductive health [16]. Hyperandrogenemia was defined as an FAI > 8.5 based on our prior work of deriving a reference value. In that study, we chose FAI values > 95th percentile among Samoan women in the lowest tertile of BMI [17].

Fasting insulin and glucose assays were performed by Northwest Lipid Labs in Seattle, WA, USA [18]. HOMA-IR was defined as (plasma insulin (µU/mL) * glucose (mg/dL))/ 405 [19]. Insulin resistance was defined as HOMA-IR > 3.80, based on a study of Mexican American women [20].

### Anthropometric and health questionnaire measures

Standard procedures for anthropometry were used [12]. BMI was categorized using body composition-defined Polynesian cutoffs for overweight (BMI 26–32 kg/m^2^) and obesity (BMI > 32 kg/m^2^). Normal and underweight categories were collapsed into one category due to the low prevalence in each group (9.10%, 0.29%, respectively).

Women were classified as having amenorrhea (AM) if they answered no to the question: ‘*Have you had a menstrual period in the last 12 months*?’. If they answered yes to that question, we then asked them to choose one of the following: having it now; within the last month; 1–3 months ago; 3–6 months ago; 6–9 months ago; or 9–12 months ago. Normal menstrual cycles were classified as having their last period within the last 3 months. Oligomenorrhea (OM) was defined as having their last period 3–12 months ago [13, 21]. Oligomenorrhea (OM) and amenorrhea (AM) groups were collapsed into one category due to the small sample in each category.

Due to the low prevalence, 24%, of use of hormonal contraception (HC) in the general population, participants with missing data (*n* = 154) were assumed to not be using HC [13]. Tobacco use was evaluated as a categorical covariate as current cigarette smokers or non-smokers [18].

Women were classified as having suspected PCOS if they had two of the following three criteria: irregular menstrual cycle (oligomenorrhea, OM; amenorrhea, AM), hyperandrogenemia define as FAI > 8.5, or an AMH above the 90th centile among those with normal FAI levels and regular menstrual cycles.

### Statistical analyses

Baseline continuous variables were described using mean and standard deviation (sd) if normally distributed, and median and log10-transformed standard deviation if not normally distributed, and by frequency and percentage tables for categorical variables. AMH values were transformed to SI units (ng/mL x 7.14 = pmol/L). Since AMH values were not normally distributed, log10 AMH values were used for most analyses, including modeling its change with cross-sectional age.

We imputed the 84 missing AMH values, 12.5% of all values, which were < LoD, 0.006 ng/mL (0.043 pmol/L) with the next theoretically measurable value of 0.005 ng/mL (0.036 pmol/L). We were guided by methodological work on the problem of values < LoD, which indicated that simple substitution with a single value had negligible effect when this occurs in < 25% of the sample [22]. Because AMH values were imputed for those below the lower limit of detection, we performed sensitivity analyses with and without the imputed AMH values. To determine normal reference ranges for serum AMH, we estimated the 90th and 95th percentiles of AMH for the sample of women with both normal FAI (≤ 8) and regular menstrual cycles (i.e., excluding those with high FAI levels > 8.5 and irregular menstrual cycles, OM/AM).

Pearson correlations were estimated to analyze the associations between untransformed and log10-transformed AMH levels and continuous variables. T-tests and ANOVA were used to detect differences in log10-transformed AMH across categorical variables. We computed AMH mean, median, 90th and 95th percentile for all women in each five-year incremented age groups: 25–29, 30–34, 35–39, 40–44, and 45-50.8 years old.

Models assessing the relationship between log10 AMH and age were evaluated, using age, age^2^ and age^3^, and the largest adjusted R^2^ was chosen as the best descriptive model. Sensitivity analysis of the relationship of age with AMH was also performed excluding those with imputed AMH levels. Models of AMH decline with age, stratified by BMI group and menstrual regularity status, were constructed. A p-value < 0.05 was considered statistically significant. All statistical analyses were performed using R [23].

## Results

A study sample of *N* = 670 was available after 349 women were excluded due to: history of reproductive surgical procedures, *n* = 33; history of hormonal contraceptive use, *n* = 256; or missing glucose and insulin measures, reproductive biomarkers, or menstrual cycle regularity information, *n* = 60. Those included were younger, with higher AMH levels, and higher FAI levels, but not different in BMI, than those excluded (Supplementary Table 1). Mean age was 37.9 *±* 7.7 years, and the median (IQR) was 38.4 (13.9) years (Table 1). Overweight or obesity was present among 90.5% of participants based on Polynesian BMI standards, and 27.2% were current tobacco smokers. Mean AMH was 13.45 pmol/L (sd 19.65) with a median of 6.01 pmol/L. The mean and median of log10 AMH were 0.48 (sd 1.01) and 0.78, respectively.

**Table 1:** Description of study sample of Samoan women, *N*= 670

Characteristics	% (*n*)	Mean (sd)/Median (IQR)/ Range
Age (yrs)		37.9 (7.7)/ 38.4 (13.9)/25.0– 50.8
Age Group (yrs) 25-29	22.1 (148)	
30-34	16.6 (111)	
35-39	17.5 (117)	
40-44	20.2 (135)	
45-50.8	23.7 (159)	
AMH (pmol/L)		13.45 (19.64)/6.01 (16.17)/0.036 -120.09
AMH Levels >90th percentile1	12.4 (83)	
AMH Levels >95th percentile1	6.7 (45)	
BMI (kg/m2)		34.6 (6.9)/33.9 (9.3)/18.0– 59.9
BMI Groups - Normal (<26 kg/m2)	9.5 (64)	
Overweight (26<=x<=32 kg/m^2^)	28.4 (190)	
Obese (>32 kg/m2)	62.1 (416)	
HOMA-IR		4.08 (4.41)/ 2.78 (3.14)/0.33- 40.76
Insulin resistance	34.6 (232)	
Current Cigarette Smoking	27.2 (182)	
OM/AM	14.6 (98)	
FAI		3.95 (4.48)/2.51 (3.39)/0.14-42.57
Hyperandrogenemia	9.0 (60)	
PCOS (using >90th percentile AMH1)	5.1 (34)	
(using >95th percentile AMH1)	3.4 (23)	

sd= standard deviation, IQR- interquartile range, AMH= anti-mullerian hormone, BMI = body mass index, HOMA-IR= homeostatic model assessment of insulin resistance, OM/AM= oligomenorrhea/amenorrhea, FAI= free androgen index, PCOS= polycystic ovary syndrome

1– among entire sample based on AMH percentiles among those with normal FAI and regular menstrual cycles

The 90th and 95th AMH percentiles among the *n* = 518 women with normal FAI and regular cycles were 31.8 pmol/L and 50.41 pmol/L, respectively. Using these two AMH percentiles derived from women with normal androgen levels and menstrual cycles, 12.4% of the entire sample had AMH levels > 90th percentile, and 6.7% had AMH levels > 95th percentile (Table 1). Two or more of three features consistent with PCOS were found in 5.1%, using the 90th percentile of AMH criterion among those with normal FAI and regular cycles, and 3.4% using the 95th percentile of AMH (Table 1). AMH was positively correlated with FAI and hyperandrogenemia, but not associated with BMI, tobacco smoking, or insulin resistance (Table 2).

**Table 2:** Bivariate analysis of AMH and log10 AMH, Samoan women. *N*=670

Variables		*AMH (pmol/L)*	log10 AMH (pmol/L)
Age (yrs)		-0.61**	-0.80**
BMI (kg/m2)		-0.04	-0.04
HOMA-IR		0.002	-0.06
FAI		0.37*	0.32*
Comparison of AMH Means (sd) with Categorical Variables			
Age groups (yrs) 25-29		33.37 (26.02)**	1.40 (0.34)**
30-34		18.74 (17.55)	1.10 (0.43)
35-39		11.56 (13.39)	0.80 (0.53)
40-44		3.78 (4.97)	0.11 (0.78)
45-50.8		0.82 (1.59)	-0.74 (0.77)
BMI Groups (kg/m^2^) Normal		14.86 (18.31)ns	0.53 (1.05)ns
Overweight		14.13 (19.25)	0.50 (1.05)
Obese		12.92 (20.04)	0.46 (0.98)
Insulin Resistance	No	13.08 (18.85)ns	0.50 (0.98)ns
	Yes	14.15 (21.09)	0.44 (1.06)
OM/AM	No	14.24 (19.55)*	0.64 (0.87)**
	Yes	8.84 (19.69)	-0.45 (1.23)
Smoking status	No	13.73 (19.26)ns	0.50 (1.00)ns
	Yes	12.70 (20.69)	0.40 (1.04)
Hyperandrogenemia	No	11.52 (17.72)**	0.40 (1.01)**
	Yes	32.98 (26.63)	1.25 (0.63)

BMI= body mass index, HOMA-IR= homeostatic model assessment of insulin resistance, FAI= free androgen index, sd= standard deviation, ns - *p*>0.05. *p<0.02, **- *p*<0.001

**Fig. 1:**
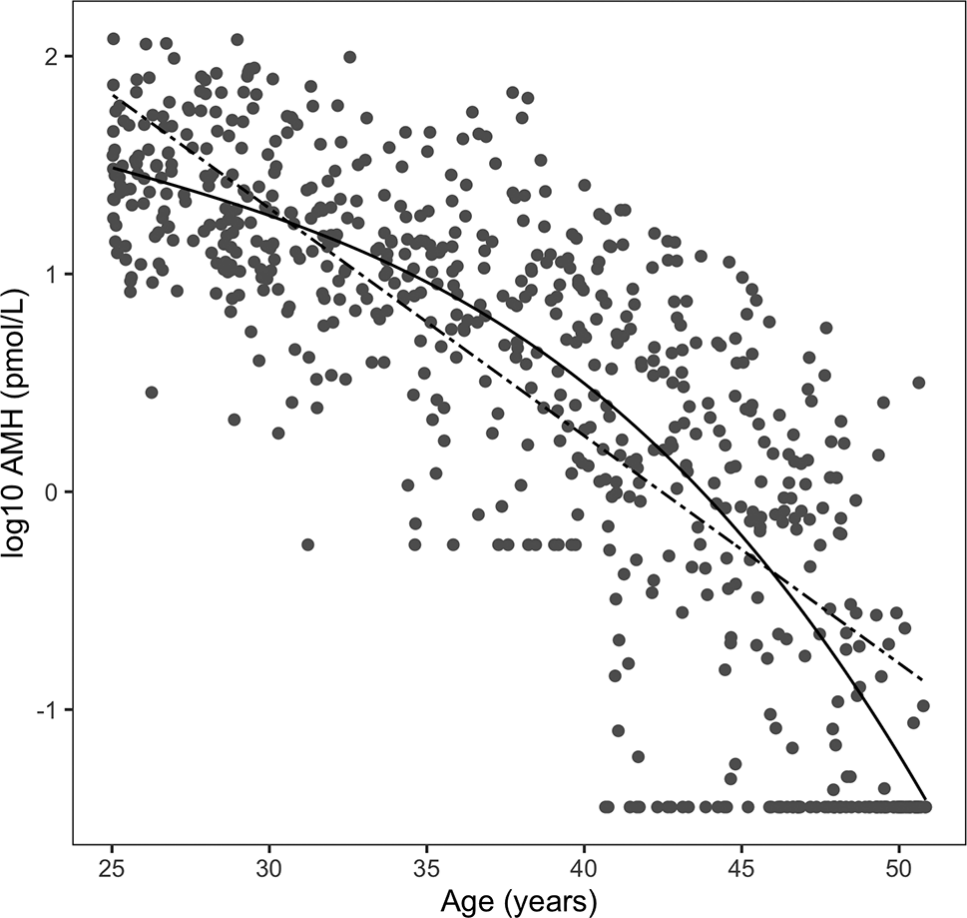
Linear (straight line) and cubic age (dashed line) models of log10 anti-mullerian hormone (AMH) levels with age. AMH rate of decline with cross-sectional age is described by the linear model: log10 AMH (pmol/L) = 4.4356–0.1045Age (R^2^ = 0.6446), and cubic model: log10 AMH (pmol/L) = 3.8943–0.^2^138Age + 0.000717Age^2^ -0.0000987Age^3^ (R2 = 0.6881)

As expected, AMH was negatively correlated with age. Linear and cubic models of log transformed AMH and age (Fig. 1) show that the cubic model best fit the data with an adjusted R^2^ value of 0.69, while the linear model had an adjusted R^2^ value of 0.64. The median, mean and 90th and 95th percentiles of AMH decreased across the five-year cross-sectional age groups (Table 3). BMI classification did not significantly affect the AMH decline with age (Supplementary Fig. 1).

**Table 3:** Anti-mullerian hormone (pmol/L) by 5-year age increments in the entire sample, *N*=670

Age group (years)	*N*	Median (Range)	Mean (log10 SD)	Age group specific
				90th, 95th percentiles
25-29	148	24.85 (2.14-120.09)	33.37 (0.34)	73.76, 84.39
30-34	111	13.35 (0.57-98.64)	18.74 (0.43)	44.55, 52.98
35-39	117	7.85 (0.57-68.04)	11.56 (0.52)	28.49, 43.98
40-44	135	1.62 (0.036-25.55)	3.78 (0.78)	11.48, 14.14
45-50.8	159	0.10 (0.036-9.64)	0.82 (0.77)	2.40, 4.13

log10 SD= standard deviation computed after logarithmic 10 transformation

Levels below the AMH assay limit of detection were imputed as 0.036 pmol/L

Sensitivity analyses found that those with imputed AMH values were significantly older, as expected, and had higher FAI levels; 10.4% of those 40–44 years had imputed AMH values, but 46.7% of those 45–51 years had imputed AMH. Excluding the imputed AMH values showed an attenuated negative association of log 10 AMH with age compared to the models with the imputed values. Without imputed AMH values R^2^ was 0.59, and the coefficients for age, -0.011, and age^2^, + 0.0003, were not as steeply negative as in the model with imputed values (Fig. 1). Sensitivity analysis contrasting those with the imputed hormonal contraception (HC) and those with measured responses to the HC questions showed no differences in other factors, except that FAI levels, but not percentage of hyperandrogenemia, were higher in those with non-imputed, i.e., measured, HC responses. More importantly, there were no differences between the two groups in the simple association of age with AMH, nor in the models using powers of age.

## Discussion

This study is the first to characterize serum AMH, define age specific AMH reference ranges, and examine associations of AMH with adiposity and reproductive factors among Samoan women ages 25–51 years. The key take home points include the following: (1) serum AMH declines in a non-linear fashion with cross sectional age; (2) among those with normal serum androgens and regular menstrual cycles, 5-year reference ranges for serum AMH were established, and the upper 90th and 95th percentiles were 31.8 pmol/L and 50.41 pmol/L, respectively; (3) AMH was not associated with BMI, insulin resistance, or smoking, (4) using two or more of three features consistent with PCOS (FAI > 8.5, OM/AM, and/or AMH at the 90th and 95th percentiles), PCOS was present in 5.1% or 3.4%, respectively. These data suggest that the serum AMH trajectories and prevalence of PCOS in Samoan women are comparable to other populations but lower than might be expected given the high prevalence of obesity in this population.

This study provides an opportunity to evaluate serum AMH in Samoan women in comparison to other populations. In Samoan women, serum AMH declined non-linearly with cross-sectional age– decreasing slowly until age 35 and then more rapidly with increasing cross-sectional age (Fig. 1). This trajectory, as well as median serum AMH levels, were similar to others [6–8]. In Samoan women with normal serum androgens and regular menstrual cycles, unadjusted linear models revealed an annual decrease in serum AMH of 9.9%. For comparison, a similar cross-sectional study of healthy American women ages 25–45 with regular menstrual cycles had annual decreases in serum AMH of 9.9%, 9.9%, 10.2%, and 6.3% among White, Latina, Chinese, and Black women, respectively [6]. Despite these similarities, data cannot be directly compared due to differences between studies in approach and/or methods for quantifying serum AMH.

AMH is secreted by granulosa cells of early ovarian antral follicles and has been implicated in PCOS pathogenesis. Serum AMH is highly correlated with antral follicle count (AFC) by ultrasound in PCOS. For this reason, serum AMH was included, for the first time, as an alternative to polycystic ovarian morphology (PCOM) by ultrasound in adults in the 2023 International Evidence-based Guidelines for the Assessment and Management of PCOS [5]. Consistent with these recommendations, a recent meta-analysis supports the use of AMH as a surrogate marker for high AFC / PCOM (pooled sensitivity of 0.79 and specificity of 0.87) as an alternative to ultrasound but not as an isolated marker for PCOS in adults [24, 25].

Nonetheless, substantial controversy remains as to the most appropriate thresholds for serum AMH in diagnosis of PCOS [24–26] since results vary by ethnic group, assay method, experimental approach, and other variables [27, 28]. A recent meta-analysis suggested diagnostic thresholds ranging from 0.81 ng/mL (5.78 pmol/mL) to 10 ng/mL (71.4 pmol/mL) based on cohort-specific data [24]. An earlier report suggested an AMH cutoff of 5.6 ng/mL (40.5 pmol/L) for PCOS [29]. A newer cutoff of 6.2 ng/mL (44.5 pmol/L), specific to the Ansh assay, was too high, missing 28.9% of patients with confirmed PCOM [28]. Since there is no universal assay or international standard for serum AMH, population- and age-specific thresholds using a specific assay are recommended.

Additional factors contribute to variability in serum AMH levels. Notably, higher BMI, characteristic of obesity, is strongly associated with PCOS but inversely associated with serum AMH [30–32]. A recent study supported a lower serum AMH threshold of 3.2 ng/mL (23 pmol/L) for BMI > 28 to achieve the same diagnostic performance (80% sensitivity and specificity) of 5.1 ng/mL (36.6 pmol/L) for BMI < 18.5 [30]. For comparison, in our study with median BMI of 33.9 (range 18.0–59.9), the 90th and 95th percentiles for serum AMH were 4.43 ng/mL (31.8 pmol/L) and 7.02 ng/mL (50.41 pmol/L), respectively. Serum AMH was not associated with BMI, insulin resistance, or smoking in Samoan women, which conflicts with published literature [8, 10]. High BMIs and low rates of smoking contribute to this finding. Inclusion of these factors did not improve the models of age and AMH (cubic age, BMI, and smoking adjusted R^2^ = 0.687; cubic age adjusted R^2^ = 0.688). Although our results are not generalizable, they may become more relevant as rates of overweight and obesity escalate globally.

Similar to the ethnic variation in serum AMH, there is also variation in prevalence of PCOS. Using two or more features (FAI > 8.5, OM/AM, and/or AMH at the 90th and 95th percentiles), PCOS was present in 5.1% or 3.4% of the population, respectively. PCOS prevalence ranges from 4 to 21% across the world [33]. A 2017 meta-analysis suggested that PCOS prevalence is lowest is in Chinese (4.4–7.3%), followed by European (4.8–6.3%), Middle Eastern (5.3–18.6%) and Black women (5.3–7.1%) [34]. Different diagnostic criteria contribute to this variation. Using only National Institutes of Health criteria, PCOS prevalence was similar (6–9%) in the United States, United Kingdom, Spain, Greece, Australia, and Mexico [35]. In addition, the risk of PCOS-associated comorbidities also varies across populations. Within the United States, Black women with PCOS have worse cardiometabolic features (i.e., HOMA-IR) than White women [3]. Additional studies are required to understand the relative contributions of genetic, cultural, racial and environmental factors to PCOS and its sequelae.

The most notable strength of our study is that it is the first of its kind in Samoan women, a population unique in its high prevalence of obesity and overweight. Furthermore, a relatively large sample size contributed to inclusive age-specific reference ranges that were subsequently used to estimate PCOS prevalence. Since ultrasound is not routinely available in Samoa, these data provide guidance on use of serum AMH as an alternative method for ovarian assessment. We also leveraged the more sensitive picoAMH assay for subjects having, or expected to have, low serum AMH. Supported by our sensitivity analysis, imputing values below the limit of detection of this assay enhanced our explanatory power and produced comparable results to age-specific trajectories in other populations [36].

Limitations of our study include the use of self-reported health information and serum biomarkers rather than formal clinical evaluation by experienced health professionals and/or ultrasound confirmation of PCOM. Subjects reported regular, irregular, or absence of menstrual periods, rather than cycle length. History of hormonal contraceptive (HC) use did not include the timing of its use, thereby necessitating exclusion of all subjects reporting HC. Included/excluded groups did not differ in BMI, HOMA-IR, or smoking, but the excluded were older and had a higher proportion of OM/AM and lower AMH and FAI (Supplementary Table 1), possibly due to use of HCs. Exclusion of subjects reporting use of hormonal contraception may have led to an underestimation of PCOS prevalence. Comparisons of PCOS prevalence across populations should be interpreted with caution due to differences in experimental approach and AMH assay methods. Lastly, further work is needed to explore the potential impact of undiagnosed PCOS and declining fertility in American Samoa and Samoa [37].

In the future, we plan to identify Polynesian-specific determinants of ovarian function across the lifespan, including genetic variation associated serum AMH using genome-wide association studies (GWAS). We have previously identified quantitative trait loci in this population with its unique history of settlement and > 2,800 years of relative isolation [38]. For example, a variant in CREBRF, rs373863828 (p.Arg457Gln), is associated with greater odds of obesity but lower odds of diabetes [38]. We will investigate the impact of this variant on measures of ovarian function and associated metabolic traits in an effort to better understand reproductive health in this population.

## Conclusions

We provide the first age-specific references ranges for serum AMH among adult Samoan women with normal serum androgens and regular menstrual cycles. We then use serum AMH as an alternative to ultrasound, in combination with FAI and OM/AM, to estimate the prevalence of PCOS in this population. This study reveals similarities (i.e., age-related decline in serum AMH, prevalence of PCOS with other ethnic groups) as well as differences (i.e., lack of association of serum AMH with BMI) between Samoans and other populations. These results provide further evidence supporting population- and age-specific thresholds for serum AMH as an alternative diagnostic criterion for PCOS, as we await more universal methods and standards. These results may facilitate earlier and/or cost-effective identification of individuals at high risk for PCOS and PCOS-associated morbidity in low-resource populations that are historically underrepresented in science and medicine.

## Electronic supplementary material

Below is the link to the electronic supplementary material.

Supplementary Material 1

Supplementary Material 2

## Data Availability

The datasets used and/or analyzed during the current study are available from the corresponding author on reasonable request.

## Abbreviations

AMH: Anti-mullerian hormone
PCOS: Polycystic ovary syndrome
BMI: Body mass index
LoD: Limit of detection
HC: Hormonal contraceptive

## References

[1] The Rotterdam ESHRE/ASRM-sponsored PCOS consensus workshop group. Revised 2003 consensus on diagnostic criteria and long-term health risks related to polycystic ovary syndrome (PCOS). Hum Reprod. 2004;19:41–7. 14688154 10.1093/humrep/deh098

[2] Anagnostis P, Tarlatzis BC, Kauffman RP. Polycystic ovarian syndrome (PCOS): Long-term metabolic consequences. Metabolism. 2018;86:33–43. 29024702 10.1016/j.metabol.2017.09.016

[3] VanHise K, Wang ET, Norris K, Azziz R, Pisarska MD, Chan JL. Racial and ethnic disparities in polycystic ovary syndrome. Fertil Steril. 2023;119:348–54. 36702345 10.1016/j.fertnstert.2023.01.031 PMC11354608

[4] Yasmin A, Roychoudhury S, Paul Choudhury A, Ahmed ABF, Dutta S, Mottola F, Verma V, Kalita JC, Kumar D, Sengupta P, et al. Polycystic ovary syndrome: an updated overview foregrounding impacts of ethnicities and geographic variations. Life. 2022;12:1974. 36556340 10.3390/life12121974 PMC9785838

[5] Teede HJ, Tay CT, Laven J, Dokras A, Moran LJ, Piltonen TT, Costello MF, Boivin J, Redman M, Boyle LA. Recommendations from the 2023 international Evidence-based guideline for the assessment and management of polycystic ovary syndrome. Fertil Steril. 2023;120:767–93. 37589624 10.1016/j.fertnstert.2023.07.025

[6] Bleil ME, Gregorich SE, Adler NE, Sternfeld B, Rosen MP, Cedars MI. Race/ethnic disparities in reproductive age: an examination of ovarian reserve estimates across four Race/ethnic groups of healthy, regularly cycling women. Fertil Steril. 2014;101:199–207. 24182412 10.1016/j.fertnstert.2013.09.015 PMC3976042

[7] Seifer DB, Golub ET, Lambert-Messerlian G, Benning L, Anastos K, Watts DH, Cohen MH, Karim R, Young MA, Minkoff H, et al. Variations in serum müllerian inhibiting substance between white, black, and Hispanic women. Fertil Steril. 2009;92:1674–8. 18930217 10.1016/j.fertnstert.2008.08.110 PMC3037722

[8] Dólleman M, Verschuren WMM, Eijkemans MJC, Dollé MET, Jansen EHJM, Broekmans FJM, Van Der Schouw YT. Reproductive and lifestyle determinants of Anti-Müllerian hormone in a large Population-based study. J Clin Endocrinol Metabolism. 2013;98:2106–15. 10.1210/jc.2012-3995 23533229

[9] Van Den Berg MH, Van Dulmen-den Broeder E, Overbeek A, Twisk JWR, Schats R, Van Leeuwen FE, Kaspers GJ, Lambalk CB. Comparison of ovarian function markers in users of hormonal contraceptives during the hormone-free interval and subsequent natural early follicular phases. Hum Reprod. 2010;25:1520–7. 20348556 10.1093/humrep/deq071

[10] La Marca A, Spada E, Grisendi V, Argento C, Papaleo E, Milani S, Volpe A. Normal serum anti-Müllerian hormone levels in the general female population and the relationship with reproductive history. Eur J Obstet Gynecol Reproductive Biology. 2012;163:180–4. 10.1016/j.ejogrb.2012.04.013 22579227

[11] Werner L, Van Der Schouw YT, De Kat AC. A systematic review of the association between modifiable lifestyle factors and Circulating anti-Müllerian hormone. Hum Reprod Update. 2024;30:262–308. 38402486 10.1093/humupd/dmae004

[12] Hawley NL, Minster RL, Weeks DE, Viali S, Reupena MS, Sun G, Cheng H, Deka R, McGarvey ST. Prevalence of adiposity and associated cardiometabolic risk factors in the Samoan genome-wide association study. Am J Hum Biol. 2014;26:491–501. 24799123 10.1002/ajhb.22553 PMC4292846

[13] Maredia H, Hawley NL, Lambert-Messerlian G, Fidow U, Reupena MS, Naseri T, McGarvey ST. Reproductive health, obesity, and cardiometabolic risk factors among Samoan women. Am J Hum Biol. 2018;30:e23106. 29663637 10.1002/ajhb.23106 PMC5980683

[14] Yildiz BO, Knochenhauer ES, Azziz R. Impact of obesity on the risk for polycystic ovary syndrome. J Clin Endocrinol Metabolism. 2008;93:162–8. 10.1210/jc.2007-1834 PMC2190739 17925334

[15] Welsh P, Smith K, Nelson SM. A single-centre evaluation of two new anti-Mullerian hormone assays and comparison with the current clinical standard assay. Hum Reprod. 2014;29:1035–41. 24578473 10.1093/humrep/deu036

[16] Bui HN, Sluss PM, Hayes FJ, Blincko S, Knol DL, Blankenstein MA, Heijboer AC. Testosterone, free testosterone, and free androgen index in women: reference intervals, biological variation, and diagnostic value in polycystic ovary syndrome. Clin Chim Acta. 2015;450:227–32. 26327459 10.1016/j.cca.2015.08.019

[17] Maredia H, Lambert-Messerlian GM, Palomaki GE, Viali S, Hawley NL, McGarvey ST. Cutoff levels for hyperandrogenemia among Samoan women: an improved methodology for deriving normative data in an obese population. Clin Biochem. 2016;49:782–6. 26908216 10.1016/j.clinbiochem.2016.02.006 PMC4915982

[18] Adia AC, Hawley NL, Naseri T, Reupena MS, McGarvey ST. Tobacco smoking patterns in Samoa in 2010: implications for interventions. Tob Prev Cessat. 2019;5. 10.18332/tpc/114093 PMC7205054 32411912

[19] Matthews DR, Hosker JP, Rudenski AS, Naylor BA, Treacher DF, Turner RC. Homeostasis model assessment: insulin resistance and ß-cell function from fasting plasma glucose and insulin concentrations in man. Diabetologia. 1985;28:412–9. 3899825 10.1007/BF00280883

[20] Qu H-Q, Li Q, Rentfro AR, Fisher-Hoch SP, McCormick JB. The Definition of Insulin Resistance Using HOMA-IR for Americans of Mexican Descent Using Machine Learning. Vella A, editor. PLoS ONE. 2011;6:e21041. 10.1371/journal.pone.0021041 PMC3114864 21695082

[21] Lambert-Messerlian G, Roberts MB, Urlacher SS, Ah-Ching J, Viali S, Urbanek M, McGarvey ST. First assessment of menstrual cycle function and reproductive endocrine status in Samoan women. Hum Reprod. 2011;26:2518–24. 21677061 10.1093/humrep/der095 PMC3157623

[22] Croghan W, Egeghy PP. Methods of dealing with values below the limit of detection using SAS carry. In: 2003.

[23] R: The R Project for Statistical Computing.

[24] van der Ham K, Laven JSE, Tay CT, Mousa A, Teede H, Louwers YV. Anti-müllerian hormone as a diagnostic biomarker for polycystic ovary syndrome and polycystic ovarian morphology: a systematic review and meta-analysis. Fertil Steril. 2024;122:727–39. 38944177 10.1016/j.fertnstert.2024.05.163

[25] Seifer DB. Elevated antimüllerian hormone level is useful in making the diagnosis of polycystic ovarian morphology and likely one day the diagnosis of polycystic ovary syndrome. Fertil Steril. 2024;122:633–4. 38977119 10.1016/j.fertnstert.2024.07.002

[26] Lie Fong S, Laven JSE, Duhamel A, Dewailly D. Polycystic ovarian morphology and the diagnosis of polycystic ovary syndrome: redefining threshold levels for follicle count and serum anti-Müllerian hormone using cluster analysis. Hum Reprod. 2017;32:1723–173. 28854584 10.1093/humrep/dex226

[27] Piltonen TT, Viita-aho J, Saarela U, Melin J, Forslund M. Utility of serum Anti-Müllerian hormone measurement as part of polycystic ovary syndrome diagnosis. Semin Reprod Med. 2024;42:049–59. 10.1055/s-0044-1786731 PMC11257749 38776986

[28] Barbagallo F, Van Der Ham K, Willemsen SP, Louwers YV, Laven JS. Age-related curves of AMH using the gen II, the PicoAMH, and the Elecsys assays in women with polycystic ovary syndrome. J Clin Endocrinol Metabolism. 2024:dgae153. 10.1210/clinem/dgae153 PMC11403310 38486510

[29] Dumont A, Robin G, Catteau-Jonard S, Dewailly D. Role of Anti-Müllerian hormone in pathophysiology, diagnosis and treatment of polycystic ovary syndrome: a review. Reprod Biol Endocrinol. 2015;13:137. 26691645 10.1186/s12958-015-0134-9 PMC4687350

[30] Zhang M, Liu X, Xu X, Li J, Bu Z, Yang Q, Shi H, Niu W, Dai S, Liang Y, et al. The reference value of anti-Müllerian hormone to diagnose polycystic ovary syndrome is inversely associated with BMI: a retrospective study. Reprod Biol Endocrinol. 2023;21:15. 36726106 10.1186/s12958-023-01064-y PMC9890853

[31] Jaswa EG, Rios JS, Cedars MI, Santoro NF, Pavone MEG, Legro RS, Huddleston HG. Increased body mass index is associated with a nondilutional reduction in antimüllerian hormone. J Clin Endocrinol Metabolism. 2020;105:3234–42. 10.1210/clinem/dgaa436 PMC7448935 32756952

[32] Lefebvre T, Dumont A, Pigny P, Dewailly D. Effect of obesity and its related metabolic factors on serum anti-Müllerian hormone concentrations in women with and without polycystic ovaries. Reprod Biomed Online. 2017;35:325–30. 28624344 10.1016/j.rbmo.2017.05.013

[33] Lizneva D, Suturina L, Walker W, Brakta S, Gavrilova-Jordan L, Azziz R. Criteria, prevalence, and phenotypes of polycystic ovary syndrome. Fertil Steril. 2016;106:6–15. 27233760 10.1016/j.fertnstert.2016.05.003

[34] Ding T, Hardiman PJ, Petersen I, Wang F-F, Qu F, Baio G. The prevalence of polycystic ovary syndrome in reproductive-aged women of different ethnicity: a systematic review and meta-analysis. Oncotarget. 2017;8:96351–8. 29221211 10.18632/oncotarget.19180 PMC5707105

[35] Wolf WM, Wattick RA, Kinkade ON, Olfert MD. Geographical prevalence of polycystic ovary syndrome as determined by region and race/ethnicity. IJERPH. 2018;15:2589. 30463276 10.3390/ijerph15112589 PMC6266413

[36] Kelsey TW, Wright P, Nelson SM, Anderson RA, Wallace WHB. A Validated Model of Serum Anti-Müllerian Hormone from Conception to Menopause. Vitzthum VJ, editor. PLoS ONE. 2011;6:e22024. 10.1371/journal.pone.0022024 PMC3137624 21789206

[37] United Nations, Department of Economic and Social Affairs, Population Division. (2022). World Population Prospects 2022, Online Edition. https://population.un.org/wpp/downloads?folder=Standard%20Projections&group=Fertility

[38] Minster RL, Hawley NL, Su C-T, Sun G, Kershaw EE, Cheng H, Buhule OD, Lin J, Reupena MS, Viali S, et al. A thrifty variant in CREBRF strongly influences body mass index in Samoans. Nat Genet. 2016;48:1049–54. 27455349 10.1038/ng.3620 PMC5069069

