# Supplementary material for: Serum anti-mullerian hormone levels and age among Samoan women
Source: Reprod Biol Endocrinol. 2025 Mar 19;23:45. doi: 10.1186/s12958-025-01379-y (PMC11921511; doi:10.1186/s12958-025-01379-y)
Supplement: Supplementary file 1 — Supplementary Material 1 [file 12958_2025_1379_MOESM1_ESM.docx]

Supplementary Table 1. Characteristics of included and excluded Samoan women ages 25-51 years (N=1,019)

Characteristics Excluded (N=349) Included (N=670) p-value

Mean Age years (Range) 42.2 (25.1 - 50.9) 37.9 (25.0 - 50.8) <0.001

Mean BMI kg/m2 (Range) 35.0 (19.2 – 59.5) 34.6 (18.0-59.9) ns

Mean AMH pmol/L (Range) 7.25 (0.36 – 183.9) 13.45 (0.036 – 120.1) <0.001

Mean Log 10 AMH pmol/L (Range) -0.04 (-1.45 – 2.26) 0.48 (-1.45 – 2.08) <0.001

Mean FAI (Range) 2.98 (0.19 – 37.73) 3.95 (0.14 – 42.57) <0.001

BMI= body mass index, AMH= anti-mullerian hormone, FAI= free androgen index
