# Supplementary figures and images for: Serum anti-mullerian hormone levels and age among Samoan women
Source: Reprod Biol Endocrinol. 2025 Mar 19;23:45. doi: 10.1186/s12958-025-01379-y (PMC11921511; doi:10.1186/s12958-025-01379-y)

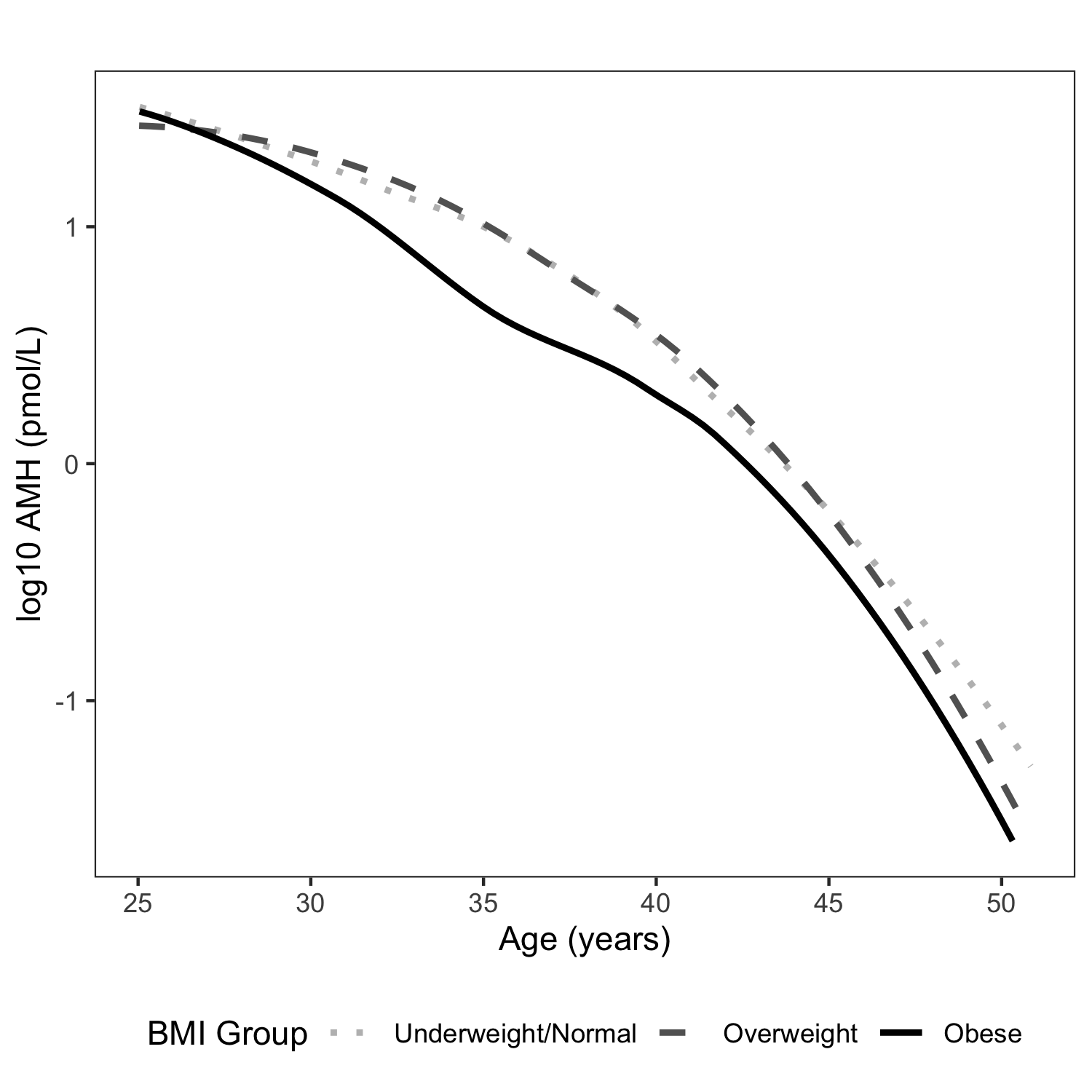

Supplement: Supplementary file 2 — Supplementary Material 2 [file 12958_2025_1379_MOESM2_ESM.tiff]
